# Boerhaave’s syndrome: A case report of damage control approach

**DOI:** 10.1016/j.ijscr.2019.04.030

**Published:** 2019-04-19

**Authors:** Sara Catarino Santos, Bruno Barbosa, Milene Sá, Júlio Constantino, Carlos Casimiro

**Affiliations:** Serviço de Cirurgia Geral do Centro Hospitalar Tondela-Viseu, Avenida Rei D. Duarte, 3504-509, Viseu, Portugal

**Keywords:** Boerhaave’s syndrome, Oesophageal perforation, Damage control, Case report

## Abstract

**Introduction:**

Boerhaave’s syndrome is a life-threatening oesophageal perforation that carries a high mortality rate (20–50%). Diagnosis is difficult by its rarity and the absence of typical symptoms. Treatment of this condition usually requires surgical intervention.

**Presentation of case:**

We report the case of a 77-year-old man that resorted to the emergency room with dyspnoea and thoracic pain after vomiting. CT scan revealed pneumomediastinum, left collapse lung and loculated pleural effusion. A left intercostal chest tube was inserted with food drainage. Hence, Boerhaave’s syndrome was suspected. Thoracotomy with mediastinum debridement, pleural drainage and oesophageal T-tube drainage was performed. Patient was admitted on the Intensive Care Unit with septic shock, with need for ventilatory support and vasopressor therapy. Two days later, a second look thoracotomy was done with definitive oesophageal repair and pleural patch. The post-operative course was complicated by pneumonia and stroke. Patient was discharged home on the 38th day and remains well at 3 month of follow-up.

**Discussion:**

Delayed diagnosis and treatment are the principal causes of high mortality in Boerhaave’s syndrome. The classic Mackler’s triad (vomiting, lower thoracic pain and subcutaneous emphysema) is present in less then 50% of cases. A thoracic drainage may be useful to confirm diagnosis promptly. There is no standard treatment option. In this case report, the authors used a damage control approach to control sepsis, allowing for a delayed definitive oesophageal repair.

**Conclusion:**

Prompt diagnosis with thoracic drainage and a damage control treatment plan might lead to good prognosis for patients with this rare and potentially fatal condition.

## Highlights

• Boerhaave’s syndrome is a rare life-threatening condition, usually requiring urgent surgical treatment.
• Thoracic drainage may confirm diagnosis rapidly.
• Debridement and drainage of pleural space and mediastinum are essential in sepsis control.
• Damage control approach with oesophageal T-tube drainage may help in sepsis control, allowing delayed definitive oesophageal repair.
• The outcome is determined by rapid diagnosis and timely and effective treatment.

## 1 Introduction

Boerhaave’s syndrome, first described by Herman Boerhaave in 1729, is a rare but potentially fatal condition characterized by a transmural oesophageal tear. This disease is caused by an abrupt rise of intraluminal pressure secondary to vomit [1]. It requires urgent diagnosis and treatment. Diagnosis is challenging, as the classic triad of Mackler (vomiting, lower thoracic pain and subcutaneous emphysema) is present in less than 50% of cases [2]. It requires a multidisciplinary management and, if delayed, severe complications may develop. The optimal therapeutic approach to this condition has not been standardized, but surgery is the cornerstone of treatment [3]. Herein, we present an unusual case and discuss the diagnostic, surgical management and outcome. The present work has been reported in line with the SCARE criteria [4].

## 2 Case report

**Fig. 1:**
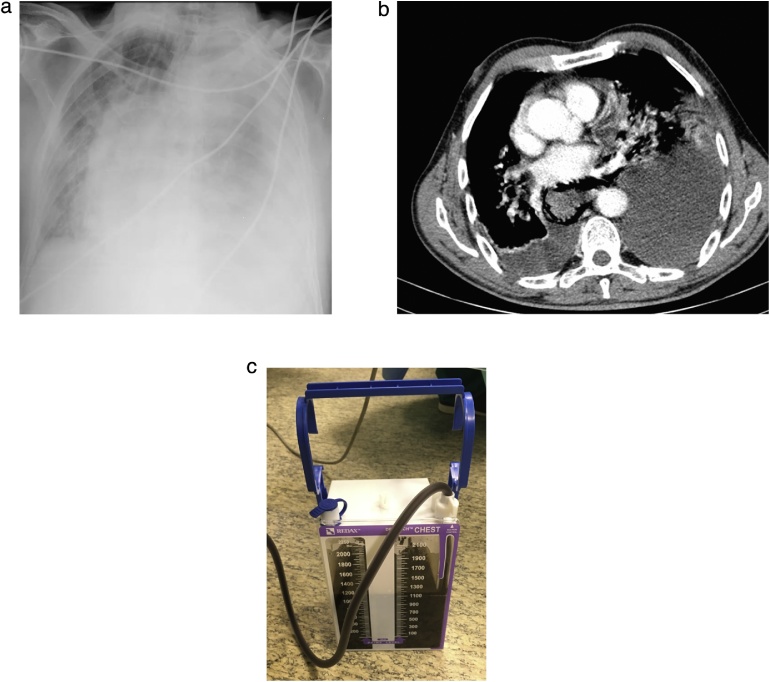
Diagnosis progress. A. Chest x-ray – left pleural effusion. B. CT scan – pneumomediastinum, left collapse lung and heterogeneous pleural effusion. C. Chest tube drainage – food residue.

**Fig. 2:**
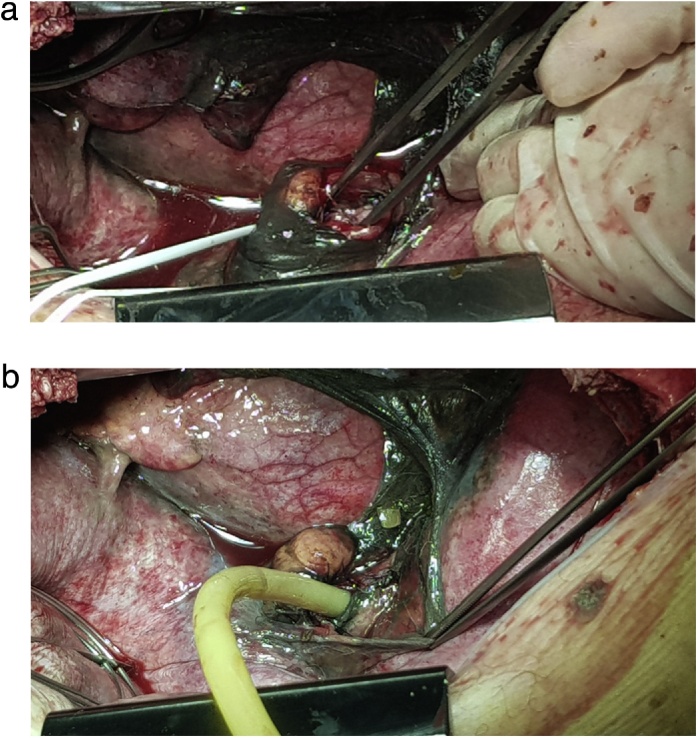
Emergent left thoracotomy. A. Debridement and drainage of the pleural space and mediastinum. B. T-tube oesophageal drainage.

**Fig. 3:**
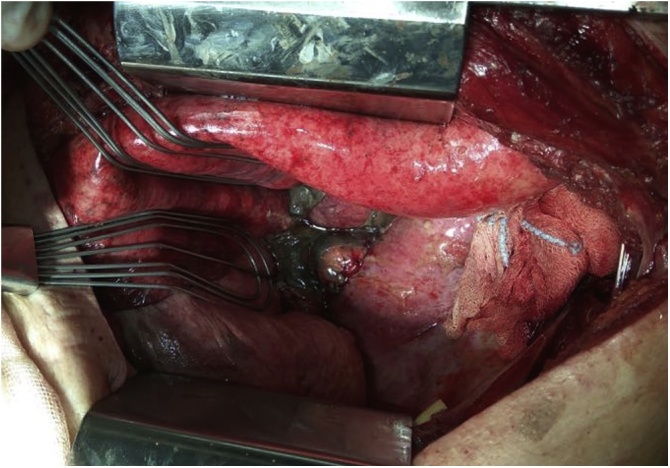
2nd look thoracotomy – oesophageal closure and pleural patch.

**Fig. 4:**
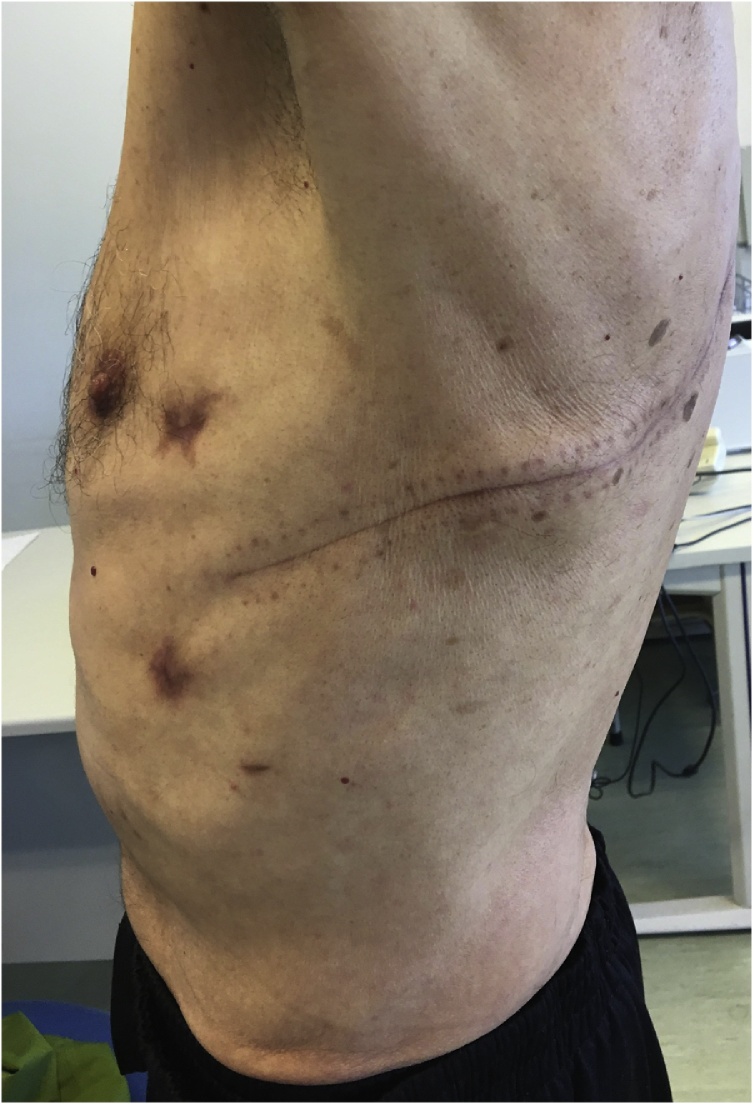
Final outcome.

The authors report the case of a 77-year-old caucasian man that presented to the emergency department with sudden onset of dyspnoea, chest retrosternal pain and epigastric pain. Complains were preceded by vigorous vomit. Patient had previous medical history of diabetes, dyslipidaemia and benign prostatic hyperplasia. Upon admission the patient was tachycardic but with normal arterial pressure and no fever. On physical examination breath sounds were diminished on the left side and there was pain in the upper abdomen. All laboratory data were within normal limits but arterial blood analysis revealed Pa_O2_ 47 torr, Sat_O2_ 78% and hyperlactacidaemia (2.7 mmol/L) on Fi_O2_ of 32%. Chest x-ray showed a large left pleural effusion (Fig. 1A). A CT scan was performed and revealed pneumomediastinum, left collapsed lung and loculated pleural effusion (Fig. 1B). A left intercostal chest tube (32 Fr) was inserted with residue food drainage (Fig. 1C). Hence, Boerhaave’s syndrome was suspected and, as the patient’s general condition was progressively deteriorating, an emergent surgery was undertaken. Patient was intubated with a double-lumen tube (direct visualization laryngoscopy). The patient was positioned in right-lateral decubitus and a left thoracotomy was performed. Intraoperatively a collapsed left lung was found with large amounts of food material (Fig. 2A) and a 2.5 cm longitudinal tear on the left-lower oesophagus was identified. The patient rapidly became more unstable, with need of vasopressor support. So, the authors decided to aggressively debride and irrigate the chest cavity. A T-tube was positioned and sutured to the oesophageal perforation (Fig. 2B) in order to create a controlled fistula. Two chest tubes were inserted and the chest wall was closed. The patient was then admitted to the Intensive Care Unit (UCI) with need for ventilatory support and vasopressor therapy. Intravenous broad-spectrum piperaciline/tazobactam antibiotherapy was initiated. Forty-eighth hours later, a second look thoracotomy was undertaken with further lavage and a definitive oesophageal repair – 3/0 monofilament interrupted sutures reinforced by using a pleural patch (Fig. 3). The remaining ICU stay was complicated by right side pneumonia, but with no prolonged ventilatory support needed. He was transferred to the general surgery ward on the 8th postoperative day. During the hospital stay the patient suffered a fall with blunt head force trauma and head CT scan showed a stroke. Antiplatelet drugs were initiated and physiotherapy was done. He was discharged home on the 38th postoperative day. On 3 months follow-up consultation, the patient is well with no impairments (Fig. 4).

## 3 Discussion

Boerhaave’s syndrome is a rare clinical entity with a mortality rate of 20–50% [5,6]. Its pathophysiology involves a sudden rise in intraluminal oesophageal pressure, most often during or after intense vomiting [1,2]. It accounts for only 15–30% of all oesophageal perforations. The others result from iatrogenic, traumatic, foreign-body and disease-related perforations [1,7]. In the majority of cases, the rupture is located in the lower third of the oesophagus, about 2–4 cm above the cardia, where there is a relative scarcity of longitudinal muscle fibers, an absence of local anatomical structural protection, and a large number of associated vascular and neural structures that weaken the oesophageal wall [2,6]. Perforations are usually longitudinal (mean of 22 mm) and the left side is more commonly affected than the right, due to an anatomical weakness of the left posterolateral aspect of the oesophagus just above the diaphragm [6].

Diagnosis of Boerhaave’s syndrome is challenging as history and symptoms can be nonspecific [2,7,8]. It is reported that there is a male predominance of 2:1–5:1 and is more frequently seen in people aged 50–70 years [9]. Common symptoms include vomiting, chest pain, dyspnoea, dysphagia, subcutaneous emphysema, tachycardia, fever, tachypnea and epigastric pain. Since these symptoms are not limited to spontaneous oesophageal perforation, they may be confused with other more common conditions as pneumonia, myocardial infarction, spontaneous pneumothorax, pulmonary embolus, aortic dissection, perforated peptic ulcer and pancreatitis [7]. Prompt diagnosis is one of the most important factors for patient’s outcome. There are several imaging tools available for diagnosis: X-ray, oesophagography, endoscopy and CT scan. However, the findings are dependable on disease duration, site of rupture and integrity of mediastinal pleural, leading to high false negative rates [6]. Besides, endoscopy may potentially aggravate the oesophageal tear [6,10]. CT scan is considered nowadays the most effective method for early detection of oesophageal perforation [6]. In this case report, the authors decided to do a thoracic drainage, which is an easy method to corroborate a suspicious diagnosis early in the course of diagnosis investigation.

Management of Boerhaave’s syndrome remains controversial and the optimal therapeutic approach has not been standardized. There are three levels of treatment: a conservative, an endoscopic and a surgical approach [1]. The choice between these treatment strategies is closely related to time interval, location, size of the tear, extent of chest contamination and patient’s clinical status [6]. Nevertheless, the principles are the same: sepsis control by limiting diffusion of contamination, adequate drainage, perforation repair and antibiotic treatment [6], because, as we know, the predominant causes of death are sepsis and multiple organ failure [9]. Most authors declare that the surgical approach remains the cornerstone of treatment for most cases of Boerhaave’s syndrome [1,2,4,6,8,11,12]. Finally, “what kind of surgical approach is the best” is another point of controversy: surgical treatment ranges from a less invasive approach consisting of debridement and drainage of the chest cavity to extensive resection of the thoracic oesophagus [1]. In this case report, the authors show a damage control approach to control sepsis in the first surgery by T-tube drainage of the oesophagus, debridement and drainage of the mediastinum and pleural cavity, followed by a delayed definitive oesophageal repair in the second surgery. The disadvantage of this approach is the need of two planned thoracotomies and prolonged ICU stay. The benefits are allowing sepsis control and stabilization of the patient’s clinical status before definitive repair, mainly in patients with extensive chest contamination. This approach may possibly prevent the need of more radical surgical interventions as oesophageal resections and may prevent the high postoperative leaks rate after primary repair, which is as high as 50% according to some studies [11]. The authors believe that this damage control treatment plan may reduce the morbidity and mortality associated with Boerhaave’s syndrome.

## 4 Conclusion

Boerhaave’s syndrome is a rare and life-threatening condition that still represents a diagnostic and therapeutic challenge. Prompt recognition and timely treatment are important prognostic determinants. Thoracic drainage may be a helpful tool to confirm diagnosis early. Treatment depends on various factors but usually requires surgical intervention. A damage control approach may be used to control sepsis initially, allowing for a delayed oesophageal repair after patient’s stabilization. This damage control treatment plan might lead to a good outcome.

## Conflicts of interest

Nothing to declare.

## Sources of funding

This research did not receive any specific grant from funding agencies in the public, commercial, or not-for-profit sectors.

## Ethical approval

Clinical case exempts from ethical approval in my institution.

## Consent

Written informed consent was obtained from the patient for publication of this case report and accompanying images. A copy of the written consent is available for review by the Editor-in-Chief of this journal on request.

## Author’s contribution

Sara Catarino Santos – data collection, data analysis and interpretation and writing the paper.

Bruno Barbosa – data collection and data analysis.

Milene Sá – operated the patient, data analysis and interpretation and writing the paper.

Júlio Constantino – data analysis and manuscript review.

Carlos Casimiro – manuscript review.

## Registration of research studies

Clinical case report, not formal research project.

## Guarantor

Milene Sá.

## Provenance and peer review

Not commissioned, externally peer-reviewed.
